# Health commodity management information system (Dagu-2 Software) implementation status in public health facilities of South-western Oromia, Ethiopia: a mixed method study

**DOI:** 10.1186/s12913-024-12199-y

**Published:** 2025-01-04

**Authors:** Tadesse Gudeta, Nimona Berhanu, Azmeraw Bekele, Bekele Boche, Tidenek Mulugeta, Gizachew Tilahun, Bodena Bayisa, Julia Kleineidam

**Affiliations:** 1 School of Pharmacy, Institute of Health, Jimma University, Jimma, Ethiopia; 2 Logistics Education (LEED) at Kühne Foundation, Hamburg, Germany

**Keywords:** Implementation status, Dagu-2, Public health facilities, South-western Oromia, Ethiopia

## Abstract

**Background:**

To ensure the complete traceability of healthcare commodities, robust end-to-end data management protocols are needed for the supply chain. In Ethiopia, digital tools like Dagu-2 are used in the lower levels of the healthcare supply chain. However, there is a lack of information regarding the implementation status, factors, and challenges of Dagu-2, as it is a recent upgrade from the offline Dagu-1 application. Thus, this study aimed to assess the implementation status of Dagu-2 in public health facilities in Southwestern Oromia, Ethiopia.

**Methods:**

The study employed a sequential explanatory mixed method design to investigate the implementation status of the Dagu2 program in 33 public health facilities in the Southwestern Oromia region of Ethiopia. Study participants were selected using a two-step approach. Firstly, public hospitals and health centers that had implemented Dagu-2 were identified. Secondly, 65 logistic practitioners, including store managers and pharmacy heads, who met the eligibility criteria were selected for the quantitative study. Quantitative data were collected using validated and reliable self-administered questionnaires and analysed using SPSS version 23. We run both descriptive and inferential statistical analyses. Fisher's exact test was used to discern the relationship between dependent and independent variables at *p* < 0.05. The qualitative data were gathered through in-depth interviews and underwent manual thematic analysis.

**Results:**

Out of 65 questionnaires, 61 were completed (93.8% response rate). About 77.0% reported using Dagu-2 for operational and strategic decisions, and 80.3% used it for logistics performance monitoring. Roughly 78.7% of the participants indicated a positive implementation status for Dagu-2. Antivirus usage (*p* = 0.018) and administrative support (*p* = 0.002) significantly associated with the implementation. External support and user-friendliness facilitated the implementation, while infrastructure constraints, connectivity absence, weak management support, and project handover gaps were major obstacles.

**Conclusion:**

Overall, the study revealed a promising implementation process and service quality improvements. However, challenges such as lack of management support, limited ICT infrastructure, absence of connectivity, weak management support, and project handover gaps became obstacles for successful implementation. To ensure an effective healthcare system, leveraging technology tools and securing stakeholder support through training are essential.

**Supplementary Information:**

The online version contains supplementary material available at 10.1186/s12913-024-12199-y.

## Background

To strengthen the healthcare system, a well-integrated supply chain is essential for ensuring the constant availability and affordability of healthcare commodities. Supply chain managers must have a comprehensive understanding of all operations, including need anticipation, procurement, inventory management, warehousing, distribution, funding, and policy implementation. Effective decision-making at each stage relies on high-quality, consistently updated data, making a robust logistics management information system (LMIS) critical [1, 2].

Information Technology (IT) provides a foundation for information sharing and collaboration throughout the supply chain. By integrating digital technology into supply chain management, organizations can enhance service quality, cost-effectiveness, efficiency, and inventory management while facilitating ongoing improvements. IT also ensures smooth access to logistics information across diverse organizations, achieving comprehensive traceability of healthcare commodities [3–5]. However, many developing countries still rely on cumbersome paper-based procedures for collecting logistics data, resulting in time-consuming processes and potential supply chain failures, such as product stock outs [6–8]. The slow adoption of modern digital infrastructure presents significant barriers to healthcare improvement, affecting care coordination and timely data integration [9].

In some African countries that have adopted digital logistics systems, challenges persist regarding system functionality. The effectiveness of LMIS has been undermined by slow processing times and inadequate data integration [10–12]. For instance, a recent report from Rwanda highlights that while logistics information systems enhance service efficiency, areas such as system integration and tracking accuracy still need attention [13]. Similarly, a study in Kenya identifies funding constraints, service prioritization, and inadequate data-sharing policies as obstacles to the successful implementation of digital systems [14].

In Ethiopia, the reporting and requesting processes for stock and commodity orders are resource-intensive and prone to errors, leading to disparities in medical product availability [15]. Limited infrastructure has hindered supply chain visibility, particularly for last-mile health facilities. As a result, public health facilities continue struggling to meet the healthcare needs of communities due to shortages of essential medicines and supplies leading to compromised service quality, and poor care outcomes, and disproportionately affects vulnerable individuals who rely on remote sites, lack resources for travel, or have chronic illnesses and no insurance [16].

To address these challenges, the Ministry of Health, in collaboration with USAID Digital Health Activity (DHA) and other partners, has implemented strategies to strengthen Ethiopia's electronic LMIS. Dagu is a widely used software solution designed to manage supply chain functions at health facilities, incorporating inventory and patient service management systems. The upgrade from Dagu 1.0 to Dagu 2.0 is anticipated to revolutionize supply chain management by enabling health facilities to manage requests electronically, automate pharmacy services, and improve data visibility, all of which are expected to significantly reduce report preparation time, inventory accuracy and medicine shortages [17, 18].

As of 2022, Dagu-2 has been extended to approximately 28.1% of health facilities in Ethiopia. The Ministry of Health, alongside partners like DHA, is actively working to expand program coverage. However, apart from a pilot survey conducted at six health institutions, there is a scarcity of empirical information regarding the implementation of Dagu-2 [19]. A more extensive survey is necessary to generalize findings and understand the current implementation status, including challenges and facilitators. Consequently, this study adopts a mixed-method approach to comprehensively explore the aforementioned issues in the southwestern region of Oromia, Ethiopia.

## Methods

### Study setting and period

**Fig. 1:**
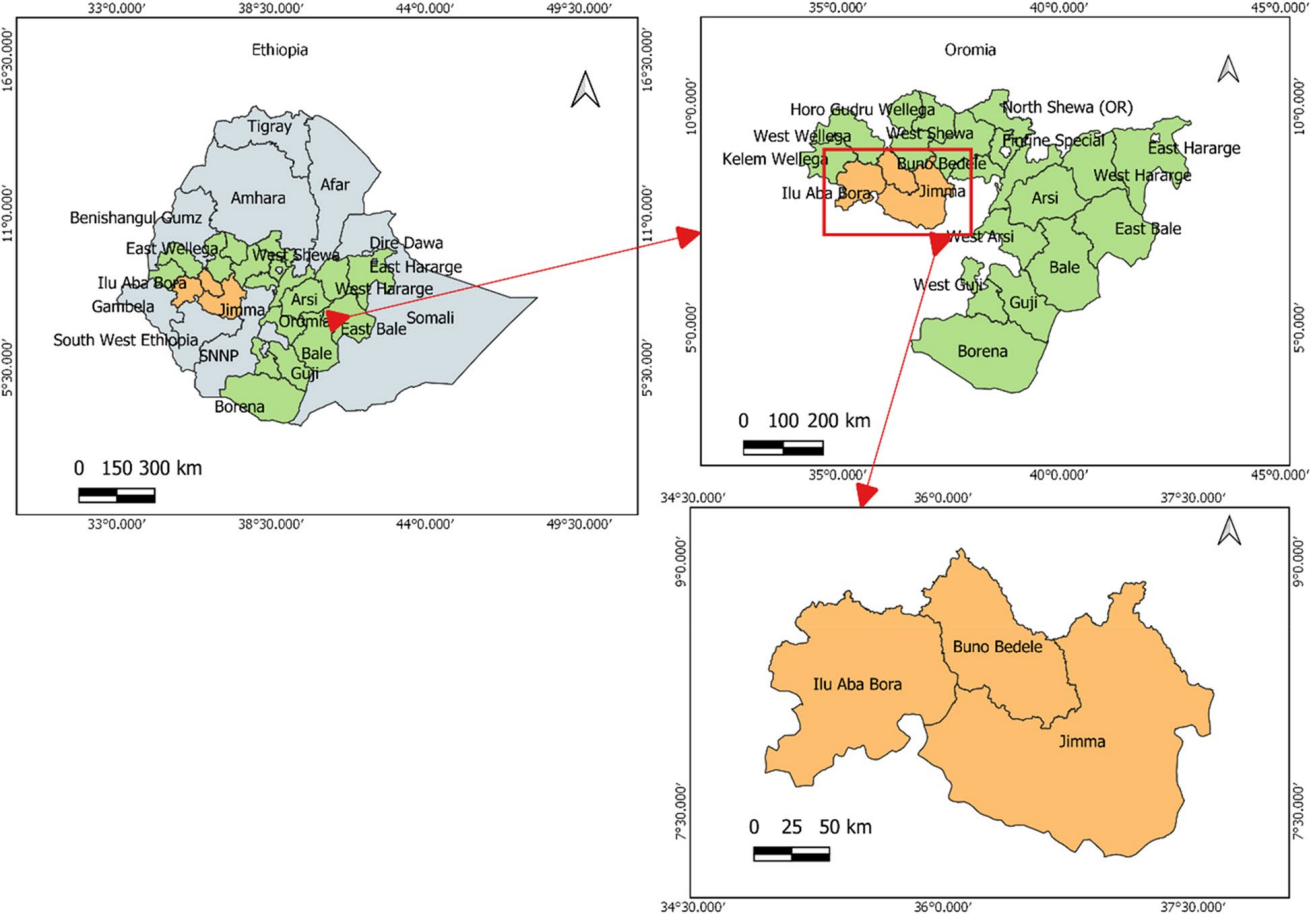
Map of the study area, October 08 to December 10, 2023

The study was conducted from October 8 to December 10, 2023, in public health facilities located in the southwestern region of Oromia, Ethiopia. This region includes Jimma zone and city, as well as the Illubabor and Bunno Bedele zones (Fig. 1). According to the 2007 report by the Central Statistical Agency (CSA), the projected population of Jimma zone and city is approximately 3,686,155 and served by nine hospitals and 125 health centers. The Illubabor zone has a projected population of 2,271,609 and has 39 health centers and two hospitals, with Metu as its capital. Bunno Bedele, established as an independent zone in 2016, has a population of 829,663 and includes three hospitals and 32 health centers. Overall, the three zones and Jimma City comprise 14 public hospitals and 196 health centers. Notably, ten hospitals and 33 health centers have implemented the Dagu-2.0 program, according to data from the Ethiopian Pharmaceutical Supply Service hub.

### Study design

We applied a sequential explanatory mixed-methods approach. This quantitative and qualitative blended approach was used to improve the explanation and validation of the quantitative data. Moreover, the transition of the program from an offline (Dagu-1) to web-based system (Dagu-2) is expected to introduce various new elements, challenges, and practices that necessitate in-depth exploration for a more comprehensive understanding. The qualitative data were subsequently triangulated during the discussion phase.

### Population

The study involved health facilities that adopted the Dagu-2 program and met the specific criteria for inclusion and exclusion stated below. It also included logistic personnel, like store personnel and pharmacy heads, who were required to regularly use the program to manage pharmaceutical transactions in their facilities and met the inclusion criteria set by the researchers. On the other hand, the source population consisted of all public health facilities in the study area that had implemented the Dagu-2 program and the respective logistic personnel (storekeepers and pharmacy heads).

### Inclusion and exclusion criteria

The study only included health facilities that had used the Dagu2.0 program for over a year and logistic practitioners who had at least one year of experience with the program. On the other hand, practitioners who were not available during the data collection period, and those who were not volunteer to participate were excluded.

### Sampling procedure

We employed a two-stage sampling method to choose the desired participants. In the first stage, we identified 33 facilities that met the inclusion and exclusion criteria from a pool of 43 Dagu-2 implementing facilities. In the subsequent phase, we selected all the respondents from the facilities identified in the first stage, encompassing a sum of 65 store managers and a pharmacy head.

For the qualitative component, we purposefully selected a group of participants consisting of four store managers, five pharmacy heads, one Dagu cluster coordinator, and a member of the supply chain team. Participants were chosen based on their experience each having at least five years in pharmacy services or logistics management information systems and their respective positions. Moreover, these individuals are anticipated to become acquainted with the Dagu-2 program through regular use in tasks such as inventory recording, transactions, and engagement in supervision and coordination activities. Thus, a total of eleven interviews were conducted. The sample size decision in this part depended on the principle of data saturation, i.e., the point in consecutive interview sessions where ideas begin to repeat. In our study, the reiterations started after the ninth interviewee.

### Data collection procedures

Various hospital staff members, including the pharmacy head, store manager, and supply chain coordinators, were questioned to collect data. Trained data collectors used pre-tested, self-administered structured questionnaires to gather quantitative data. These questionnaires were divided into three parts: the first part collected socio-demographic information, the second part focused on facility characteristics (such as infrastructure, technical issues, and managerial aspects), and the third part addressed the implementation status of DAGU and software-related factors. We used Yes/No questions and a five-point Likert scale (ranging from 1, denoting strongly disagree, to 5, implying strongly agree) to address parts II and III.

The researchers used semi-structured interview guides that contained probing questions to delve into the qualitative data. To ensure consistency, investigators moderated discussions and recorded and took notes of interviews that lasted approximately 45 min on average. We used the local languages, including Afan Oromo and Amharic, on the preferences of the interviewees to make communication easier.

### Data processing and analysis

The data collection formats were checked manually for completeness and consistency of responses before coding and entering the variables into the software. Subsequently, the quantitative data were analyzed using Statistical Package for the Social Sciences (SPSS) software version 23 in descriptive statistics, including means, frequencies, and percentages. The results were presented in frequency tables and graphs. We conducted Fisher's exact test to determine the relationship between Dagu-2 implementation status and related factors. The Likert scale agreement responses were recoded to simplify analysis. Consequently, "Strongly disagree," "disagree," and "neutral" were consolidated into "disagree," while "strongly agree" and "agree" were merged into "agree".

In contrast, the qualitative data were analyzed manually using the thematic analysis technique. After transcribing and translating the recordings into English, the researchers assigned codes to variables and organized them in a tabular format. We grouped similar codes to establish relevant themes. These themes were then described, and the perspectives of certain interviewees were quoted to highlight the severity of the issues.

### Data quality assurance

We performed data quality checks before, during, and after the data collection process, to maintain the validity and reliability of our instruments. Data collectors received one hour of training on data collection approaches, and field supervisions were conducted for proper data collection. Data collection formats were designed after extensive literature reviews and face validated by an expert affiliated with a Kuhne foundation. A pre-test was conducted at a health center in the Jimma zone (later excluded from the actual study) to standardize instruments and ensure error-free forms.

Concerning the qualitative assessment, researchers were well-acquainted with the subject matter and moderated the discussions. In-depth interviews were conducted with participants possessing relevant expertise to ensure the data's credibility. The methodological descriptions guaranteed the study's transferability and dependability, while an explanation of the whole research process further enhanced the trustworthiness of the results.

### Operational definitions

#### System quality

System quality in this study pertains to the software's user-friendliness, adaptability, efficiency, consistency in operation, and possession of essential syntaxes.

#### Southwest Oromia

The study focused on the southwest Oromia region, which included three zones (Jimma, Ilu Aba Bor, and Bunno Bedele) and one city administration (Jimma City Administration).

## Results

### Socio-demographic characteristics

Sixty-five questionnaires were distributed to Dagu 2 software users, with 61 duly completed, resulting in a 93.8% response rate. Of the participants, 47 (77.0%) were males, and most of the respondents, 48 (78.7%), fell within the age range of 26–35 years. Forty-three (70.5%) participants had a bachelor's degree, and all respondents were pharmacy professionals. The notable portion of the participants, 45 (73.8%), were hospital staff. Most of those practitioners, 41(67.2%), haven’t received Dagu-2 training. Moreover, 42 individuals (68.9%) had a service experience of five years or more. The qualitative study included eleven participants, aged 28 to 40, each with over five years of service. The group consisted of individuals in diverse roles, and were predominantly male (Table 1).

**Table 1:** Sociodemographic characteristics of respondents in public health of Southwest Oromia, Ethiopia. October 08 to December 10, 2023

Quantitative study		
Variables	Items	Frequency (%)
Gender	Male	47 (77.0)
	Female	14 (23.0)
Age (in years)	18–25 years	4 (6.6)
	26–35 years	48 (78.7)
	36–45 years	9 (14.8)
Level of education	Diploma	18 (29.5)
	BSc/B.Pharm degree	43 (70.5)
Profession	Pharmacy	61 (100)
Working place	Hospital	45 (73.8)
	Health Center	16 (26.2)
Dagu2 training	Received	20 (32.8)
	Not received	41(67.2)
Work experience	< 5 years	19 (31.1)
	≥ 5 years	42 (68.9)
Qualitative study		
Gender	Male	7
	Female	4
Age (in years)	28–40	11
Level of education	Diploma	4
	BSc/B.Pharm degree	7
Profession	Pharmacy	10
	IT expert^a^	1
Working place	Hospital	6
	Health Center	4
	Other^a^	1
Position	Store manager	4
	Pharmacy head	5
	Dagu cluster coordinator	1
	Supply chain team member	1
Experience	≥ 5 years	11

^a^*USAID* Dagu cluster coordinator of Southwest Oromia

### Status and support analysis of the Dagu 2 program

Table 2 illustrates findings on the status of Dagu-2 implementation and also summary of the key program system qualities, available technical and administrative support, and the IT infrastructure and resources.

**Table 2:** Status and support analysis of the Dagu 2 program among public health facilities in Southwest Oromia, Ethiopia. October 08 to December 10, 2023

Variables	Questions/items	Agree (%)	Disagree (%)
Dagu utilization/implementation	Relied on Dagu-2 information for operational and strategic decisions	47 (77.0)	14 (23.0)
	Relied on Dagu-2 information to make performance monitoring	49 (80.3)	12 (19.7)
	Overall implementation	48 (78.7)	13 (21.3)
System quality	No much failure during operation	49 (80.3)	12 (19.7)
	User friendliness	48 (78.7)	13 (21.3)
	Adaptability of the software	42 (68.9)	19 (31.1)
	The software has sufficient key syntaxes	58 (95.1)	3 (4.9)
	The software improves efficiency	57 (93.4)	4 (6.6)
Administrative support	Allocation of sufficient budget	36 (59.0)	25 (41.0)
	Staffing sufficient professionals	30 (49.2)	31 (50.8)
	Regular supportive supervision	22 (36.1)	39 (63.9)
IT technical support and infrastructure	Timely provision of technical support	24 (39.3)	37 (60.7)
	Adequacy of technical assistance	12 (19.7)	49 (80.3)
	Competency of technical support providers	47 (77.0)	14 (23.0)
	The technical support improved staff ability	48 (78.7)	13 (21.3)
	A sustainable power supply	40 (65.6)	21 (34.4)
		Yes (%)	No (%)
	Any anti-virus uses to protect the system?	28 (45.9)	33 (54.1)
	Power back-up to protect interruptions?	34 (55.7)	27 (44.3)
	Use manual record along with the software?	54 (88.5)	7 (11.5)

#### Implementation status of the Dagu 2 program

Concerning the implementation status, 47 participants (77%) affirmed that health facilities make use of the Dagu-2 program for operational and strategic decisions. Similarly, approximately 80% of the surveyed individuals indicated agreement that their facilities employ software reports to monitor their logistics performance. The overall implementation status of the program within the health facilities was rated to be 78.7%.

#### Dagu2 system quality

The study requested participants to rate various aspects of the Dagu software system quality. Forty-nine individuals (80.3%) affirmed no interruptions or system failures in the software program, while 48 participants (78.7%) found the software user-friendly. Additionally, a significant number of respondents, 58 (95.1%), agreed that the software had adequate essential functionalities. Moreover, 93.4% of the respondents agreed that the software contributed to service efficiency.

#### Technical and administrative supports

Concerning the technical support, 49 participants (80.3%) expressed disagreement regarding the sufficiency of the support provided. However, 47 participants (77.0%) rated that the technical assistance providers have adequate competency, and 48 participants (78.7%) agreed that the support enhanced staff capabilities. Regarding administrative support, 39 respondents (63.9%) showed disagreement with the presence of regular supportive supervision. Moreover, approximately 59% of the respondents, agreed that budget allocations for various expenses associated with the Dagu 2 program were sufficient. However, close to 50% of the respondents contested the adequacy of staffing in terms of professional expertise.

#### IT infrastructures and support systems

Regarding support systems, 33 individuals (54.1%) reported that their facilities do not use antivirus software to ensure the smooth operation of the Dagu system. However, 34 participants (55.7%) reported having a power backup system for electrical outages. Moreover, 54 respondents (88.5%) indicated that they use a manual recording system alongside the Dagu software. About 66% of the participants agreed with the presence of a sustainable electric power supply.

### Inferential statistical analysis

Upon conducting the chi-square analysis applying Fisher's exact test, it was discovered that only antivirus usage (*p* = 0.018) and administrative support (*p* = 0.002) exhibited significant association with the implementation status of the Dagu-2 software at a two-sided significance level*.* For the remaining variables, including technical and administrative support, system quality, power supply, practitioners' level of education and experience, as well as Dagu-2 training, the results indicate a non-significant association (*p* > 0.05) with Dagu-2 implementation (Table 3).

**Table 3:** Factors associated with implementation status of Dagu-2 among public health facilities in Southwest Oromia, Ethiopia. October 08 to December 10, 2023

Variables		Implementation status		Fisher's exact test	
		Poor (%)	Good (%)	Sig (2-sided)	Sig (1-sided)
Data security (Use of anti-virus)	Yes	13 (46.4)	15 (53.6)	0.026^*^	0.018^*^
	No	27 (81.8)	6 (18.2)		
Technical support	Disagree	15 (40.5)	22 (59.5)	0.088	0.044^*^
	Agree	4(16.7)	20 (83.3)		
Administrative support	Disagree	18 (42.9)	24(57.1)	0.003^*^	0.002^*^
	Agree	1(5.3)	18 (94.7)		
System quality	Disagree	6(26.1)	17(73.9)	0.578	0.356
	Agree	13(34.2)	25(65.8)		
Power supply	Disagree	6(28.6)	15 (71.4)	1.000	0.495
	Agree	13(32.5)	27(67.5)		
Power backup	Yes	11(32.4)	23(67.6)	1.000	0.522
	No	8(29.6)	19(70.4)		
Manual system	Yes	19(35.2)	35(64.8)	0.088	0.062
	No	0(0.0)	7(100)		
Level of education	Diploma	4(22.2)	14(77.8)	0.381	0.255
	BSc/B.Pharm	15(34.9)	28(65.1)		
Ownership	Hospital	16 (35.6)	29 (64.4)	.346	.177
	Health Center	3 (18.8)	13 (81.2)		
Dagu2 training	Yes	6(30.0)	14(70.0)	1.000	0.568
	No	13(31.7)	28(68.3)		
Work experience	< 5 years	3(15.8)	16(84.2)	0.135	0.071
	≥ 5 years	16(38.1)	26(61.9)		

^*^Significant at *p* < 0.05

## Qualitative results

### Description of the participants

The interviewees shed light on various issues concerning the practices, facilitators, and challenges related to the implementation of Dagu-2 within the context of the LMIS.

### The LMIS practices

The interviewees described the pharmaceutical recording and reporting systems of their facilities, initiatives taken to enhance information systems, preparations made before the implementation of Dagu-2, and an overview of the current status of Dagu-2.

#### Pharmaceutical recording and reporting systems

The recording and reporting system employed in public health facilities combines both traditional manual methods of paper-based recording and reporting, as well as the utilization of digital applications. In certain health centers, the manual system remains the predominant approach for recording and reporting. For instance, one of the pharmacy heads mentioned,

*"Currently, we do not use any software to manage our inventory; we have no active computers in the dispensing units or the store. We handle all our activities using hard copy formats such as bin-cards, IFRR, and RRF."* (33-years old, male, health center pharmacy head).

Previously, the digital application Dagu-1 was utilized, and it has now been updated to the web-based electronic logistics management information system Dagu-2, which is currently being used alongside the manual system. One individual stated,

*"Before Dagu-2, we used Dagu-1. Later, we upgraded to Dagu-2, which is a significant improvement. It allows us to track prices and stock status (it has its own bin and stock cards), which were missing in Dagu-1."* (29 years old, female, health center store manager).

The simultaneous use of both the digital application and the manual system consumes more time for personnel and reduces work efficiency. The adoption of this dual approach is due to legal ramifications from the Ministry of Finance during financial audits.

#### Efforts made to improve the recording and reporting systems

Participants discussed various initiatives aimed at enhancing LMIS and inventory management. The government in collaboration with partners introduced digital applications like Dagu-1 and Dagu-2, along with the auditable pharmaceutical transaction system (APTS), to upgrade LMIS from a manual to a digital system. One of the pharmacy store managers stated that,

"*Previously, we managed our inventory manually, but since last year we have transitioned to a computerized system, such as using Dagu 2.*" (31-year-old, male, health center store manager).

#### Preparations made to implement Dagu-2

The implementation of the DAGU-2 application in public health facilities lacked proper planning, leading to variations in experiences among the participating facilities. The accounts provided by the participants present conflicting narratives. When asked about the preparations made, such as professional training, one participant responded,

*"No, there was not. We received implementation instructions directly from higher levels"* (32 years old, male, hospital pharmacy head).

In contrast, another participant from a different facility reported preparatory work, including professional training, conducted before the application's implementation. He stated,

*"First, we received training, and then I took over. We practiced it as a trial phase for one month before attempting full implementation"* (31 years old, male, hospital store manager).

#### Current status of the application

The health facilities are using the application parallel to the manual system in most of the health facilities for recording every health commodity transaction within the health facility and generating inventory reports. One of the respondents replied,

*“For recording and inventory report generating, we use Dagu 2 software. It is comprehensive. Parallel to that, we also have hard copy formats (models) like model 22.”* (28 years old, female, health center store manager).

Most health facilities print the inventory report known as RRF from the application and send it to concerned bodies. Printing hard copy was required because the facilities are not connected to the local area network (LAN) of the application, had the health facilities' computers been connected, it would have been possible to directly send their request and report to a supplier called Ethiopian pharmaceutical supply and services (EPSS). However, the application is not in utilization in some of the health facilities, especially the health centers.

“*To be honest, Dagu is not active in our health facility at this time; I remember that it was started around three years ago, and failed without sustaining*." (33 years old, male, health center pharmacy head).

The reasons for the failure mentioned by the participants are shortage of human resources that caused excessive workload which is not compensated, lack of commitment of the staff, lack of support from the administration, and lack of formal training on the applications are the major reasons mentioned.

#### The use of the manual system alongside the digital application

The intention behind the digital application was to eliminate the reliance on manual paper-based recording and reporting. However, in all health facilities, there was a parallel use of the manual system alongside the digital application. The simultaneous use of both the manual and digital systems created a double burden for those working in the store and the Logistic Management Information System unit. The participants cited legal consequences during financial auditing as the primary reason for not completely abandoning the manual system. One participant explained,

"*We use both. For example, we use the manual Model 19 for receiving items. The auditing system itself encourages paper-based reports. The reports generated by Dagu-2 are not acceptable for internal and external audits, which obliges us to use paper-based reports."* (31 years old, male, hospital pharmacy store manager).

Another reason for utilizing both systems relates to the limitations of the application itself. Participants mentioned that the application does not include a comprehensive list of all medicines used in public health facilities, making it difficult to add these products to the application. Moreover, the absence of a separate requisition form, known as Model 20, used by health facilities in Ethiopia, was raised as an issue. One of the pharmacy heads explained,

*"We use both. There are items (products) outside the Dagu list. We manage those items manually. The system lacks Model 20 (requesting format). It only generates Model 19 and 22,"* (35 years old, female, hospital pharmacy head).

The utilization of multiple recording and reporting formats by different stakeholders is identified as the fundamental issue, particularly the lack of alignment between the formats used by the Ministry of Finance and those used by the Ministry of Health (MOH) and Ethiopian Pharmaceutical Supply Service (EPSS). Harmonizing these formats would address the problem at its core.

### Facilitators of the Dagu-2 implementation

The interviewees highlighted that the support from external organizations and the user-friendliness of the program facilitated the implementation of Dagu-2.

#### Support from external bodies

The participants mentioned that one of the facilitators in the implementation of Dagu-2 was the material support received from NGOs to enhance the ICT infrastructure of public health facilities. One store manager stated,

"*We have received support in the form of computers and Code-Division Multiple Access (CDMA) from USAID."* (30-year-old, male, hospital store manager).

Having an up-to-date ICT infrastructure is crucial for implementing digital solutions in healthcare facilities. Strengthening the ICT infrastructure through support has a positive impact on implementing digital solutions. Another form of support mentioned in this category was the supportive supervision provided by various governmental and non-governmental organizations. One of the participants explained,

*"USAID mostly helps us technically; EPSS, Oromia Regional Health Bureau, and sometimes MOH supervise us on how we are practicing with it."* (34 years old, male hospital store manager).

Supportive supervision allows for monitoring the implementation status of different interventions, identifying areas of weakness, and providing technical support and on-the-job training to professionals. It is particularly important in the early phases of implementing various interventions. Additionally, having a designated focal person from EPSS available for any required technical support in health facilities was also identified as a facilitator in implementing the DAGU-2 application in public health facilities.

#### User friendliness of the application

Digital applications aimed at modernizing information management systems in healthcare facilities should possess user-friendly interfaces, be easily comprehensible, simple to operate, and allow users to address minor technical issues effortlessly. An essential factor that facilitates the implementation of Dagu-2 in public health facilities is the application's quality in terms of user-friendliness. For instance, one of the respondents stated,

*"From my perspective, as a pharmacy professional, every element of the tool is transparent and understandable. It is straightforward, even for individuals with basic computer skills."* (32 years old, male, hospital pharmacy head).

The application's quality of user-friendliness serves as a crucial determinant of its success or failure during implementation. Alongside the aforementioned facilitators, the presence of supportive supervision from facility management, the application's significance in enhancing data quality, its ability to secure data, the availability of trained personnel during implementation, and the potential for interconnectivity with EPSS hubs are also highlighted as facilitators for the implementation of Dagu-2 in healthcare facilities.

### Challenges of Dagu-2 implementation

This study identified five major Dagu-2 implementation challenges in public health facilities, including problems related to infrastructure, data visibility, technical issues of the application, management support, and project handover.

#### Infrastructure related

The implementation of digital solutions for logistics information management systems necessitates investments in ICT infrastructures. These infrastructures typically include high-capacity computers, local area networks (LAN), backup servers, and internet connections, which need to be available at the facilities. Many participants expressed concerns regarding issues with computers, internet connections, and local area networks, attributing these problems as reasons for the difficulties. One participant stated,

"*Internally, we lack functioning computers. It is challenging to implement any initiative that requires ICT infrastructure*." (28 years old, female, health center store manager). This sentiment was reiterated by another participant who mentioned similar issues with ICT infrastructure. He said, "*We cannot fully implement the Dagu 2 system due to our outdated computer, which uses Windows seven and processes slowly. Sometimes, we are unable to open the system*." (30-year-old, male, hospital store manager). Both participants highlighted the challenges posed by the lack of proper ICT infrastructure in their respective settings.

These problems were also noted in the observation field note, where it was observed that most computers were very old, resulting in longer loading times for applications and slower navigation. The situation indicates a lack of appropriate planning before implementing the application in health facilities or a lack of attention from facility management regarding the importance of ICT infrastructure.

#### Lack of interconnectivity

The Dagu-2 application, a web-based tool, is designed to provide access to all computers connected to the network. Its purpose is to enhance data visibility within health facilities and extend to EPSS hubs located outside the facilities. The program offers time-saving features such as balance review, identification of near-expiry items (within 6 months), and stock status tracking. However, in practical observations, it was noted that the computers in almost all health facilities were not connected to the network. Consequently, the pharmacy head is unable to monitor the stock status in the store, and EPSS managers cannot oversee the stock status in the health facilities within their catchment area. One of the store managers expressed this lack of connectivity, stating,

"*It is not yet interconnected. The communication between the store manager and pharmacy head by itself is not connected. We both use a single computer with two separate user names*." (30 years old, male, hospital pharmacy store manager).

Another participant, one of the participants also highlighted the issue, stating,

*"The other concern is that the application is not directly connected to EPSS. If it were connected, it would allow data visibility, enabling EPSS to monitor the situation in health facilities."* (34 years old, female, health center pharmacy store manager).

#### Weak management support

The successful implementation of any initiatives relies on the close monitoring and support of managers, whether they are within or outside the facility in the healthcare system. Many participants highlighted that facility management does not pay attention to the information management system, resulting in a lack of response to problems and solutions proposed by the pharmacy team. The participants believed that the management did not comprehend the system's importance. One participant expressed his concern by stating,

"*The big challenge related to management is their lack of understanding of the system's importance, leading them to not consider it as a significant tool*." (33 years old, male, health center pharmacy head).

This lack of attention from management may stem from issues during the planning phase of implementation. It appears that the health facilities' management was not involved in the planning and implementation of the system.

For example, one crucial form of support is hiring an adequate number of well-trained human resources in the facilities. Many participants raised concerns about the insufficient number of trained professionals in the facilities. One of the pharmacy store managers mentioned,

"*The lack of adequate human resources is a problem. For instance, if I encounter a social issue, the store activities would come to a halt, or they would have to relocate pharmacists from the dispensary unit.*" (30 years old, male, hospital store manager).

Additionally, another significant issue related to human resources is the lack of commitment among professionals.

#### Technical issues of the application

The software development process is a continuous and ongoing endeavor, aimed at providing updates to address user issues and implementing major changes as necessary. The Dagu-2 application, despite its usefulness, has encountered some technical problems highlighted by participants in the study. One significant issue raised pertains to handling errors during data entry. One of the pharmacy heads expressed the problem as follows,

“*If an error is made in one batch, it necessitates voiding all data associated with that product, including data from other batches with similar names*.” (35 years old, female, hospital pharmacy head).

To prevent the initiative from failing, the application developers must gather user feedback and make necessary updates to address this problem, as the frustration caused by the application may lead to its failure.

#### Gaps in project handover

The Dagu program system was initiated under the USAID delivery project, which aimed to provide essential resources and support systems such as computers, printers, Interrupted Power Supply (UPS), and training on IPLS and Dagu for the practitioners. Over time, the number of facilities increased and received continuous support. However, challenges arose during the project handover from one support provider to the new partner during the phase-out period. The Dagu-2 cluster coordinator emphasized the issue, stating that.

“*The phase-out process of various USAID projects, including the delivery project (lasting five years), the AIDS-free project (lasting three years), and the current DH project (lasting five years), resulted in new partners having to start from scratch. Unfortunately, the handover of the project from one partner to another was not smooth, which hurt the progress of system implementation and led to performance falling short of expectations. Additionally, there is a deficiency in supportive supervision on the government's part*.” (40 years old, Male, Dagu-2 cluster coordinator).

## Discussions

This study evaluated the implementation status of Dagu-2 in public health facilities. The key points addressed in this study were: the implementation status of Dagu-2 factors associated with the implementation of Dagu-2, facilitators, and challenges of Dagu-2 implementation in public health facilities.

### Implementation status of the Dagu-2 program

Recorded evidence shows that using technological solutions such as Dagu-2 minimizes errors, avoids redundancy, facilitates informed decisions in supply chain management, and enhances overall customer service [6, 20]. According to the current study, 78.8% of health facilities have implemented the program. According to the qualitative evidence, almost all interviewed individuals revealed that they have been using both the data management system (Dagu-2) and paper-based activities due to failure to fully implement the program.

The interviewed concerned bodies revealed that Dagu-2 was not fully implemented due to many factors like lack of training, lack of ICT infrastructure, lack of supportive supervision, and so on. Additionally, the qualitative evidence explored that the system was implemented without thorough situation analysis and preparedness, which affected its sustainability. Apart from poor preparedness, the negative attitude of the management team of health facilities was another thing that affected the sustainability of the Dagu-2 implementation.

### Dagu2 system quality

This study assessed the quality of the Dagu-2 tool by examining factors such as operational failures, user-friendliness, software adaptability, presence of key software syntaxes, and contribution to operational efficiency. The findings revealed that 80.3% of the participants agreed that there were no interruptions during system operation. Additionally, 78.7% of the participants found the application to be user-friendly. Moreover, 95.1% of the participants agreed that the software had key syntaxes. Furthermore, 93.4% of the participants agreed that Dagu-2 enhanced system efficiency. We can understand from the study that the overall quality of the system is worth sustainable implementation and scale-up to other health facilities.

Previous studies also evidenced that the usage of information technology significantly improves operational performance. For instance, the study conducted in India reported the performance improvement of supply chain activities ranged from 9 to 41% after the implementation of the Information Communication Technology tool along the supply chain echelon [16]. Moreover, the study conducted in Pakistan uncovered that the implementation of information technology tools in supply chain management improves the quality of service by facilitating informed decisions throughout the supply chain echelon [21]. Furthermore, research conducted in Tanzania and Zambia highlighted the substantial impact of eLMIS utilization on service quality by simplifying data collection processes and streamlining the reporting and requisition of medical products at the end of each month [21].

The impact of using Information Communication Technology on performance efficiency is also evidenced by the study conducted in Kenya, which revealed that the tool enhanced inventory management techniques, improved communication with internal and external customers, and enhancement of order processing [22]. Even though the current study did not objectively measure the impact of Dagu-2 implementation using baseline data and post-implementation data, the participants reported that they had witnessed the difference in quality of service and simplification of data processing for decision-making.

The qualitative evidence in this study showed that the implementation of the program improved the quality of service and simplified many tasks, which streamlined inventory management, and order processing, and eventually improved customer service. Despite these factors, the health facilities where this program was implemented did not transition away from paper-based processes to fully adopt the program, leading to increased workloads for healthcare workers. This situation caused dissatisfaction among healthcare workers, who perceived the program implementation as an additional burden rather than a simplification of their tasks. Interviewed stakeholders cited several reasons for the concurrent use of both the Dagu-2 program and traditional inventory management practices, including concerns about program sustainability, unreliable ICT infrastructure, manpower shortages, and high turnover rates of trained personnel. In addition to the aforementioned reasons, the discrepancy of formats used by different stakeholders like the Ministry of Health, Ministry of Finance, and Ethiopian Pharmaceuticals Supply Service forced the health facilities to implement the program parallel without stopping the traditional paperwork. Therefore, in addition, to improving the infrastructure and training manpower, it is also mandatory to harmonize the formats to fully scale up and implement the inventory management tool.

### Opportunities and challenges

In this study, more than two-thirds of the participants (77%) reported that the technical assistance providers have adequate competence. Moreover, 78.7% of participants said that technical support enhanced staff capabilities. Study participants as the facilitators or opportunities to sustain the program also mentioned budget adequacy, the presence of power backup to prevent interruptions, presence of continuous power supply. For instance, 59% of participants reported that the budget allocated for Dagu-2-related activities was sufficient while 66% of participants responded that they had a sustainable power supply, which is vital for the operation of the program. Insufficiency of technical support (80.3%), absence of regular supportive supervision (63.9%), inadequacy of trained staff (50%), and absence of antivirus software on computers (54.1%) were mentioned as challenges of Dagu-2 implementation.

The limited studies available on this topic also mentioned the same challenges during the implementation of digital technologies in the health system. For instance, the scoping review that evaluated the articles published between 2015 and 2020 mentioned policy, governance complexities, and resource gaps as the challenges of implementation. Moreover, the same study listed unsustainable and fragmented systems, infrastructure gaps, and insufficient workforce capacity as the challenges [23].

Another study conducted in Swaziland mentioned capacity building of the health workers through training, supervision, and mentoring as the facilitators and backbone of LMIS implementation [24]. This finding aligns with the present study, which investigated the significance of capacity-building initiatives such as supportive supervision as facilitators playing a significant role in the sustainable implementation of Dagu-2.

The study conducted in eastern Ethiopia also mentioned inadequate power backup (64.3%), unsustainable internet connection (43%), and insufficient training (34.6%) as the challenges encountered during the implementation of the District Health Information System (DHIS) (40%). The participants of the current study also reported the aforementioned challenges with slight differences in magnitude. Though the previous study investigated the challenges encountered during DHIS implementation, it is important to consider and plan to mitigate the same challenges in the implementation of Dagu-2 since both systems need trained manpower, ICT infrastructure, Internet connection to synchronize data, and continuous supportive supervision. Furthermore, a systematic review conducted in Ethiopia aimed at identifying challenges associated with Information Communication Technology implementation revealed obstacles including a negative attitude towards technology use, lack of trained workforce, insufficient knowledge, limited training opportunities, low computer literacy, restricted computer access, poor internet connectivity, and inadequate experience with ICT hindering technology adoption [25]. From the current study and previous studies conducted in Ethiopia and abroad, we learned that the challenges encountered during digital technology implementation in healthcare are more or less similar with different intensities. Based on these collective findings, developing a robust plan and implementing challenge mitigation strategies are essential steps to ensure a successful system implementation, rather than rushing the process.

The qualitative finding also explored many facilitators and challenges related to Dagu-2 implementation. For instance, The ICT infrastructure support from partners was mentioned as the facilitator of the program implementation. The fact that many health facilities have access to electric power, the infrastructure aid from partners can facilitate the implementation and scale-up of the program. Partners like USIAD have been playing a significant role in digitalizing health systems including pharmaceutical inventory management in Ethiopia through material and technical support. To use these good opportunities, the government must transfer ownership of the system to maintain its continuity and momentum. Even though the supportive supervision was not regular and not strong, it was mentioned as one facilitator for the successful implementation. For the successful implementation of any digital technology, understandability (user-friendliness), and quality in terms of improving data visibility are very important. These factors were mentioned as facilitators of the system implementation and its sustainability. One of the qualities of Dagu-2 is its ability to enhance data visibility; for instance, the system can show the stock status of health facilities to the resupplying institution (EPSS), which facilitates decision-making and avoids overstocking, understocking, or product expiration.

Regarding the challenges explored through qualitative inquiry, poor infrastructure, weak management support, lack of interconnectivity, instability of the application, and the project handing over issues were strongly raised as the barriers encountered during the implementation process. The infrastructure was mentioned as both an opportunity and a challenge in this study by participants. This is mainly because primarily, the health facilities received infrastructure support from USAID at the start-up stage (opportunity), but have not received equivalent support from the government. Moreover, the donated ICT infrastructure did not receive repair services post-receipt (challenge or barrier). In addition to ICT infrastructure, the software (Dagu-2) needs high processor computer to be functional without becoming sluggish. Contrary to this fact, many health facilities in which Dagu-2 was implemented owned old computers with low storage capacity and low RAM that hinder the application from operating efficiently. Regarding the challenges with interconnectivity, the current study explored that the computers were not connected to internet service, which limited the data visibility in the facilities and with EPSS. While the system was intended to share data with other computers within the facilities and with EPSS and external health facilities, this functionality was not achieved in practice. This was primarily due to the absence of internet connectivity and limitations within the application that hindered data visibility among health facilities. Furthermore, the application failed to display EPSS data such as product availability, stock status, and other inventory information, which could have simplified the order processing for health facilities.

Lack of management team support for the implementation process was mentioned as another issue by interviewees in this study. The implementation process faced hurdles due to a lack of trained manpower and capacity-building training. This challenge was exacerbated by a lack of motivation from the management team to recruit extra staff and facilitate technical training for frontline users of the application. Another issue that negatively affected the implementation process was the instability of the application due to ongoing modification of the application by its developers to make it easy and user-friendly. Since the application's frustration may contribute to the initiative's failure, the developers should gather user feedback and make the necessary updates to address this issue.

Another issue that hampered the initiative's implementation and continuity was the gap created when support was transferred from one partner to another. If the handover process of an initiative from one partner to another partner is not smooth, the continuity and adaptability of the initiative are not certain. Furthermore, a lack of government support for the initiative hampered implementation and frustrated system users. To achieve national implementation, integration, and scaling of the practice, it is critical to conduct a thorough situational analysis and reach an agreement on continuous support from all stakeholders, including partners, the government, and health facilities.

## Conclusion and limitations

In conclusion, this study demonstrates that the implementation of Dagu-2 is promising, with participants reporting improvements in service quality. However, several challenges remain, including inadequate management support, limited ICT infrastructure, insufficient training, and lack of government ownership. To maximize the benefits of Dagu-2, it is critical to leverage information technology, focus on user-friendly design, and ensure strong stakeholder support and training. Management must also address the shortage of trained personnel and collaborate with partners to resolve infrastructural issues. Additionally, a comprehensive situational analysis prior to implementation can help mitigate risks, and continuous supervisory support and capacity-building training are essential for sustained success. Achieving consensus among stakeholders before transferring support to government partners is crucial.

This study has limitations, notably its lack of assessment of the impacts of Dagu-2 on pharmaceutical availability and wastage. Future research should include comparative studies at regional and national levels to gain a deeper understanding of implementation status, challenges, and opportunities, while incorporating objective measures from health facilities, customers, and suppliers (EPSS).

## Supplementary Information

Supplementary Material 1.

## Data Availability

All data supporting the findings of this study are available within the paper.

## Abbreviations

AI: Artificial Intelligence
CDMA: Code Division Multiple Access
CSA: Central Statistical Agency
DHA: Digital Health Activity
DT: Digital Technology
e-APTS: Electronic Auditable Pharmaceutical Transaction System
e-HCMIS: Electronic Health Commodity Management Information System
e-LMIS: Electronic Logistics Management Information System
ERP: Enterprise Resource Planning
HIS: Health Information System
HMIS: Health Management Information System
ICT: Information Communication Technology
IFRR: Internal Facility Report and Requisition Form
IoT: Internet of Things
IPLS: Integrated Pharmaceutical Logistics System
IRB: Institutional Review Board
IT: Information Technology
JSI: John Snow.Inc
JU: Jimma University
LAN: Local Area Network
MoH: Ministry of Health
MSD: Medical Store Department
PHFs: Public Health Facilities
RRF: Report and Requisition Form
SDPs: Service Delivery Points
SPSS: Statistical Package for Social Science
UPS: Interrupted Power Supply
USAID: United States Agency for International Development
