# Supplementary material for: Health commodity management information system (Dagu-2 Software) implementation status in public health facilities of South-western Oromia, Ethiopia: a mixed method study
Source: BMC Health Serv Res. 2025 Jan 4;25:22. doi: 10.1186/s12913-024-12199-y (PMC11699636; doi:10.1186/s12913-024-12199-y)
Supplement: Supplementary file 1 — Supplementary Material 1. [file 12913_2024_12199_MOESM1_ESM.docx]

# **Supplementary file. Data collection tools**

1. **Respondent’s profile**

1.1. Gender: **1.** Male
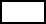
 **2**. Female
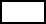


1.2. What is your age in a year? _________________________

1.3.What is your highest level of education? **1**. Diploma **2**.BSC/BA degree
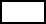


**3**. MSc/MA
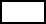
 **4**. Others; **please specify it**___________________________

1.4. What is your profession? **1**. Pharmacy profession
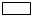
 **2**.Nurse
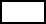
 **3**. Medical Laboratory Technology
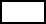
 **4**. Others; **please specify it**___________________________

1.5.What are your work experiences at your current position (in years)?__________________________

1.6. What is your working experience at this facility (in years)? ____________________________

1.7. Types of facility

1. Hospital
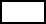
 2. Health center
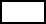


2. Please indicate your level of agreement regarding questions 2.1 to 2.4, which pertain to the quality of the Dagu system, administrative and IT technical support, IT infrastructures and support systems, and the implementation status of Dagu-2. Use the scale provided below to rate your agreement:

1 = Strongly Disagree, 2 = Disagree, 3 = Neutral, 4 = Agree, 5 = Strongly Agree

However, the last three questions in section 2.4 require a "Yes" or "No" response. Please mark "Yes" if the practice exists or "No" if it does not exist.

- 1. **Questions on DAGU2.0 system quality**

| **DAGU2.0 system quality** | **Code for items** | **Measurement items** | **1** | **2** | **3** | **4** | **5** |
| --- | --- | --- | --- | --- | --- | --- | --- |
|  | **SI1** | Dagu-2 is a user-friendly program |  |  |  |  |  |
|  | **SI2** | Dagu2.0 is reliable (i.e., not much failure during operation). |  |  |  |  |  |
|  | **SI3** | Dagu2.0 can be adapted to a variety of user needs and changing conditions. |  |  |  |  |  |
|  | **SI4** | The Dagu2.0 has sufficient syntaxes to carry out the logistics transactions (Effectiveness). |  |  |  |  |  |
|  | **SI5** | Dagu2.0 improves efficiency by reducing losses due to overstock and expiry (Efficiency). |  |  |  |  |  |

- 1. **Administrative and IT technical support for Dagu-2 implementation**

| IT-technical support | TS1 | The IT technical support provision is adequate (can be accessed through face to face, toll 8773 free line, telephone...). |  |  |  |  |  |
| --- | --- | --- | --- | --- | --- | --- | --- |
|  | TS2 | The support is provided timely for all Dagu2.0 queries and requests. |  |  |  |  |  |
|  | TS3 | The technical support providers are competent enough in the Dagu-2 |  |  |  |  |  |
|  | TS4 | The technical support improved the staff's skills in Dagu-2 |  |  |  |  |  |
| Administrative support | AS1 | Your facility provides and plans a sufficient budget for the consumable resources (paper, cartridge, UPS, and generator) needed to operate the system. |  |  |  |  |  |
|  | AS2 | Your facility allocates sufficient professionals to achieve the goal |  |  |  |  |  |
|  | AS3 | Your facility provides regular supportive supervision |  |  |  |  |  |

- 1. **Questions on ICT infrastructure and support systems**

| **Measurement** | **Code** | **Measurement items** | **1** | **2** | **3** | **4** | **5** |
| --- | --- | --- | --- | --- | --- | --- | --- |
|  | **ITI1** | The facility has a sustainable electric power supply. |  |  |  |  |  |
| Please mark "Yes" if the practice exists or "No" if it does not exist. | | | | | | | |
| Questions | | | Yes | | No | | |
|  | **ITI2** | Does your facility use a manual record and reporting system along with the application (Dagu-2)? |  | |  | | |
|  | **ITI3** | Does your facility have a power backup to protect power interruptions? |  | |  | | |
|  | **ITI4** | Do you or your colleague use antivirus to protect your computer system? |  | |  | | |

- 1. **Questions on Dagu-2 implementation status**

| **measurement** | **Code** | **Measurement items** | **1** | **2** | **3** | **4** | **5** |
| --- | --- | --- | --- | --- | --- | --- | --- |
|  | **I1** | Your facility uses system reports and information to make operational and strategic decisions. |  |  |  |  |  |
|  | **I2** | Your facility uses system reports and information for logistics performance monitoring |  |  |  |  |  |

**Interview guide: In-depth interview**

**Project tile:** The implementation status of DAGU-2 in public health facilities of Southwestern Oromia, Ethiopia: A mixed method study

**Basic information of the study**

**Setting:** Hospital/Health center

**Date:** _________________ **Venue**: ________________________

**Interviewer/s:** _____________________________

**Background information of participant:**

**Sex: ____________ Age: ___________________**

**Profession: ___________________Qualification: _____________**

**Role/Position: _________________Experience (in year): ___________**

Thanks, now we would like to start our discussions about DAGU-2.0 implementation using the following topic guide.

**Interview Questions**

| **Topic questions** | **Key questions** | **Probes** |
| --- | --- | --- |
| **Introductory questions:** Experiences of pharmaceutical recording and generating inventory reports. | | |
| 1. **A.** Experiences in pharmaceutical recording and generating inventory reports. | 1. Can you explain or brief on pharmaceutical recording and generating inventory reports in your hospital/health center? | How you record pharmaceuticals (manual, computer…)  How do you prepare inventory reports? (any software in use, ) |
|  | 1. Please briefly share with me what you know about DAGU-2.0 | Its uses, when adapted..., supported by… |
|  | 1. What efforts were ever made to improve and standardize the quality of pharmaceutical recording and reporting systems? | What initiatives are ever taken to improve pharmaceutical recording (inventory management system)? |
|  | 1. In general, to what extent are new ideas used to improve the pharmaceutical inventory management system in your health facility? | To what extent do hospital/health center administrators respond to initiatives, e.g. DAGU, LMIS…? |
| **Main questions:** We are going to talk about perceptions and experiences about the implementation of DAGU-2.0 in your health facility. | | |
| 1. **B**. Innovation domain and current status of DAGU-2.0 | Has your health facility fully implemented DAGU-2.0? | - How are you implementing it - **trial** vs **completely implemented?** - **Parallel (manual vs digital registration) or DAGU-2.0 alone?** - How was it designed? Wed based/m-based? - **Why and how** did your health facility decide to implement it? - Did you have **evidence of effectiveness**, **feasibility**, and **adoption?** - To what extent DAGU-2.0 is needed (**relative advantage compared to past experiences**) to be adopted and implemented? - To what extent DAGU-2.0 is **interconnected or networked** with other settings like EPSS, other facilities, Health bureaus, and so on? - To what extent DAGU-2.0 **is easy to use or user-friendly?** |
| 1. **C.** Impact of DAGU-2.0 implementation on health products management | •What is your opinion regarding the impact of DAGU-2.0 implementation on health commodity management? | - To what extent does it improve availability, minimize wastage, save resources (time and financial resources), and ease the process of ordering and reporting….. |
| 1. **D.** Facilitators of implementation | What are the internal (health facility) related enablers or facilitators in the process of DAGU-2.0 implementation? | - ICT infrastructure like computers, Internet, and so on - Trained manpower, facility setting or room adequacy, electricity, - Staff commitment - Management support or ownership spirit, incentives… |
|  | What are the external enablers or facilitators in the process of DAGU-2.0 implementation? | - Policy and guidelines support, Supportive supervision, Capacity building training, |
| 1. **E.** Challenges of the implementation process | What are the internal (health facility) related challenges or facilitators in the process of DAGU-2.0 implementation? | - ICT infrastructure like computers, Internet, and so on - Lack of trained manpower, facility setting, or room inadequacy - Power interruption, management-related challenges, workload, data security issues, - Lack of commitment |
| 1. **F.** Concluding and Summarizing questions | - What recommendation would you give to improve the challenges related to DAGU-2.0 implementation? - Do you have any idea to add regarding the implementation of DAGU-2.0 in this health facility that we did not mention in this interview? | |

**Thanks for your thoughtful responses and feedback!**
